# Psychosocial and environmental determinants of physical activity in a Brazilian public university employees - ELDAF: A prospective cohort study protocol

**DOI:** 10.1371/journal.pone.0263385

**Published:** 2022-02-07

**Authors:** Aldair J. Oliveira, Claudia S. Lopes, Geraldo de Albuquerque Maranhão Neto, Gustavo Mota de Sousa, Vitor Paravidino, Mikael Rostila, Mauro Felippe Felix Mediano, Rosane Harter Griep, Wesley Souza do Vale, Fabiane Frota da Rocha Morgado

**Affiliations:** 1 Laboratory of Social Dimensions Applied to Physical Activity and Sport (LABSAFE), Rural Federal University of Rio de Janeiro, Seropédica, Brazil; 2 Department of Epidemiology, Institute of Social Medicine, State University of Rio de Janeiro (UERJ), Rio de Janeiro, Brazil; 3 International Clinical Research Center (ICRC), St Anne’s University Hospital Brno (FNUSA), Brno, Czech Republic; 4 Department of Public Health Sciences, Stockholm University, Stockholm, Sweden; 5 Centre for Health Equity Studies (CHESS), Stockholm University/Karolinska Institutet, Stockholm, Sweden; 6 Evandro Chagas National Institute of Infectious Disease, Oswaldo Cruz Foundation, Rio de Janeiro, RJ, Brazil; 7 Laboratory of Health and Environment Education, Oswaldo Cruz Institute, Fiocruz, Rio de Janeiro, Brazil; University of Tampere, FINLAND

## Abstract

**Background:**

Increased physical activity levels and their determinations are essential issues worldwide. The Longitudinal Study of Physical Activity Determinants (ELDAF) aims to understand the roles of psychosocial and environmental factors in workers’ physical activity levels.

**Methods:**

A prospective cohort study of non-faculty civil servants from a public university (approximately 1,200 individuals) will start in 2022 (baseline). The primary measurements will be accelerometer- and questionnaire-based physical activity, social support, social network, socioeconomic status, bereavement, job stress, body image, common mental disorders, depression, and neighborhood satisfaction. Additional measurements will include necessary sociodemographic, physical morbidity, lifestyle and anthropometric information. Participants’ places of residence will be geocoded using complete addresses. All participants will furnish written, informed consent before the beginning of the study. Pilot studies were performed to identify and correct potential problems in the data collection instruments and procedures. ELDAF will be the first cohort study conducted in Latin America to investigate physical activity and its determinants.

## Introduction

Low physical activity levels are related to increased risk of death [1]. Physically active individuals are at lower risk of stroke [2], coronary heart disease [3], hypertension [4], depression [5], and some types of cancer [6]. Despite the known benefits, most of the world’s population does not reach acceptable physical activity levels suggested by health institutions such as the World Health Organization [7, 8]. A study with 52,936 individuals in 29 European countries reported that 40% did not engage in sufficient moderate to vigorous physical activity per week [7]. Most workers do not exceed rest levels in their occupational activities and do not include leisure activities in their daily routines [9]. In the vast majority of countries, increasing physical activity levels in the working population has been a major challenge [10].

Psychosocial factors such as stress, depression, anxiety, and body image dissatisfaction were associated with negative health behaviors (e.g. smoke, alcohol consumption and physical inactivity) [11, 12]. On the other hand, exposure to positive psychosocial factors such as social support has been related to higher levels physical activity [13]. Neighborhood environment factors are another important aspect of determinants of physical activity: neighborhood satisfaction, the built environment, and green areas are related to physical activity [14, 15].

Social support, stress, sociodemographic factors, built environment, and green spaces form part of a set of psychosocial and environmental factors that are being investigated as determinants of physical activity levels [13, 16]. However, it is only in the last ten years that epidemiological research has begun to evaluate other constructs, such as bereavement, body image dissatisfaction, job stress, and neighborhood environment satisfaction and its relationship to physical activity [17–19]. The scientific framework for these is thus smaller and further clarification of their respective roles in physical activity in needed.

In the light of the concept of social determinants of health [20], epidemiological studies have contributed significantly to understanding the links between psychosocial and environmental factors and physical activity. Inevitably, one of the most significant challenges in the field is the measurement of physical activity, because many findings are based on subjective measures. In contrast, the Longitudinal Study of the Determinants of Physical Activity (ELDAF) will use a combination of objective and subjective measurements. This approach will provide the opportunity to investigate the relationships between different physical activity dimensions (types of physical activity, moderate to vigorous physical activity, frequency of physical activity, walking behavior, active transportation) and psychosocial/environmental factors. In this regard, the ELDAF study could provide more substantial findings by virtue of four characteristics: (1) the study population comprises mainly middle-aged individuals with high prevalence of the most commons non-communicable diseases (e.g., hypertension, diabetes, overweight). In this respect, accurate health information and an understanding of the relationships between psychosocial determinants and physical activity level could be useful to the institution in implementing a health promotion program for these workers; (2) ELDAF includes a broad range of workers (blue- and white-collar workers), warranting the inference that future findings may achieve appropriate levels of external validity; (3) part of the ELDAF population lives in a rural area. Considering that scientific information on physical activity determinants in rural populations are scarce, the study will naturally contribute to the literature; and (4) follow-up starting right after a public health crisis that has had considerable impact on various aspects of people’s lives worldwide, including lifestyle and work conditions, may be significant, given that recent studies have shown worsening indicators of both lifestyle and occupational health [21, 22].

The study design–a prospective cohort study in a population of public university employees with tenure–is particularly appropriate for this type of population, particularly by facilitating follow-up. Moreover, the focus on these workers’ physical activity and its determinants is relevant, because public university employees’ working conditions have deteriorated in recent years, with low salaries and overwork as no new vacancies are opened [23]. In this regard, these factors produce more significant job stress, depression, anxiety, and other harmful conditions, which may influence physical activity levels.

This report provides broad information on the study protocol for ELDAF, a Longitudinal Study of the Determinants of Physical Activity among Brazilian public University employees from Rio de Janeiro State, Brazil. ELDAF will be the first cohort study in Latin America to evaluate solely the role of different psychosocial and environmental aspects in workers’ physical activity levels.

## Methods

### Study design

This prospective cohort study of non-faculty civil servants from a public university will take place in the Baixada Fluminense, a low-lying region of the Rio de Janeiro Metropolitan Area. The Baixada Fluminense covers approximately 2,512 km^2^ and has a population of 2,423,141, representing 40% of the population of the metropolitan area. The baseline will be in 2022, with the first follow-up two years later, and a further two waves at three-year intervals. Although the study population of approximately 1,200 does not represent all job categories in a public-sector organization, it does comprise a wide variety, including manual workers. The ELDAF population will exclude candidates: (1) assigned to another institution, (2) on leave, or (3) lacking the cognitive potential to respond to the data collection instruments.

### Data collection

Preparation for fieldwork will include accessing and reviewing lists of residential addresses and telephone numbers to contact, if necessary. All researchers participating in the fieldwork will receive intensive training in data collection. The technical administrative staff will be invited initially by letter sent to their home addresses, followed by a visit from the fieldwork assistant to their place of work and later scheduling for participation. A trained fieldwork assistant will support participants in responding to the questionnaire, if necessary. In a second visit, anthropometric, accelerometric, and blood pressure measurements will be taken. Table 1 provides more details about data collection in each phase.

**Table 1:** Description of information include according to each ELDAF phases.

	Timing of evaluation			
	Baseline—2022	2-year follow-up	5-year follow-up	8-year follow-up
Social support for PA	✓	✓	✓	✓
Social capital	✓	✓	✓	✓
Depression	✓	✓	✓	✓
Common mental disorders	✓	✓	✓	✓
Bereavement	✓			✓
Physical morbidity	✓	✓	✓	✓
Body image	✓	✓	✓	✓
Job stress	✓	✓	✓	✓
Smoking	✓	✓	✓	✓
Alcohol consumption	✓	✓	✓	✓
Eating behavior	✓	✓	✓	✓
Physical activity	✓	✓	✓	✓
Individual additional measures^£^	✓	✓	✓	✓
Public parks and squares^β^	✓			✓
Green areas^β^	✓			✓

^£^Sociodemographic information’s (age, sex, race, and marital status), sedentary behavior, body weight and stature, waist circumference, blood pressure, motivation on and tolerance in physical exercise, participation physical education class.

^β^Information will be updated after 2 and 5-years follow-up if the participant’s address change.

### Outcome measures

#### Accelerometer-based physical activity

Accelerometer-based Physical Activity Physical activity will be assessed using triaxial accelerometers (ActiGraph GT3x-BT, Pensacola, FL, USA). The device will be positioned on the non-dominant wrist to improve wear compliance [24]. Participants will be instructed not to remove the device during seven consecutive days, except during bathing and water activities. Accelerometers will be initialized to collect data in 30-Hz time resolution and 60-s epochs. Non-wear time will be set as ≥ 60 min of zero counts, with a minimum of 10 hours per awake period constituting a valid day. For each participant, days failing to provide a minimum of 10 hours of valid data will be excluded from the analysis. Weekly summary estimates of physical activity and sedentary behavior will be averaged for each participant with at least four valid days. Accelerometers will be programmed and data downloaded using ActiLife software, version 6.11.7 and the raw data will be analyzed with R-package GGIR [25].

#### Questionnaire-based physical activity

Physical activity levels will be measured using the extended version of the International Physical Activity Questionnaire (IPAQ), validated for the Brazilian population [26]. The data will be summarized as (1) total activity, reported as metabolic equivalents throughout the previous week (METs × min/week) and (2) IPAQ-defined low, moderate, or high activity categories. The high category is defined as three or more days of vigorous activity, totaling at least 1500 METs × min/week, or seven or more days of moderate physical activity, totaling at least 3000 METs × min/week. The data will also be investigated across the different dimensions of physical activity.

## Exposure measurements

### Individual measurements

#### Social support–physical activity and Social Support Scale

The Physical Activity and Social Support Scale (Brazilian version–EASAF) will be used. This instrument is based on the scale suggested by Sallis et al. [27] and was developed and validated for Brazilian adults [28]. Reproducibility testing returned alpha internal consistency between 0.87 and 0.91 and intraclass correlation coefficient between 0.63 and 0.80 [28]. The instrument consists of two blocks of questions relating to the source of social support (family and friends) for physical activities in two dimensions (walking and moderately to vigorously intense physical activities); e.g., “*In the past three months*, *how often has someone who lives with you* … *(who sleeps and has meals in the same house) taken a walk with you*? *Invited you to walk*? *Encouraged you to walk*?”. The response options are “*Never*”, “*Sometimes*” and “*Always*”. Social support scores are given by the sum of responses in each block, which range from 0 to 6 points for walking or moderately to vigorously intense physical activity.

#### Socioeconomic status

Two self-reported Socioeconomic status indicators will be included: household income and education. Household income will be calculated by dividing total monthly household income by the square root of the number of members in the household (adjusting for sex and 5-year age stratum). Participants will be categorized into four household income quartiles (upper, middle, middle-low, and low). Education will be assessed by the question, “*What is the highest graduation diploma you gained from school*?” (college, high school, middle school, elementary school). Monthly per capita family income will be calculated as total family income divided by the number of family members living on that income and then categorized in Brazilian minimum wages.

#### Bereavement

Four types of bereavement will be evaluated as loss of sibling, parent, spouse, or child. Loss of a sibling will be recorded using a dichotomous variable (Yes or No). Loss of parents will be recorded as either none, father, mother, or both. For all losses, month and year of the event will be reported. Time elapsed since loss of parents will also be recorded as: ‘‘none”, ‘‘three years ago or less, ‘‘four to eight years ago”, ‘‘more than eight years ago”.

*Job stress–Demand Control-Support Questionnaire*. Job stress will be measured using the Demand Control-Support Questionnaire proposed by Theorell and adapted to Brazilian Portuguese [29]. The DCSQ consists of 17 items distributed in 3 subscales: psychological demands, control, and social support at work. Internal consistency values (Cronbach’s alpha) ranged from 0.63 to 0.86. The scores on the first two subscales is the sum of the item scores, based on four response options ranging from ‘often’ (4 points) to ‘never/almost never” (1 point). The scores will be dichotomized at the median (demand < 15 and control < 17). Participants will be classified by Karasek quadrants: 1) high strain (high demand and low control), 2) passive job (low demand and control), 3) active job (high demand and control), and 4) low strain (low demand and high control).

#### Social network

Social network will be investigated on the modified Berkman-Syme index. This index consists of seven questions on the following: number of relatives and close friends that the respondent feels comfortable talking about almost anything with, and whom he or has contacted at least once a month and frequency of their participation in group activities, such as religious services, sports and artistic activities, and meetings of residents and employees’ associations, in the prior twelve months.

#### Body image–Body Appreciation Scale

The Body Appreciation Scale evaluates body appreciation, an aspect of Positive Body Image. The Brazilian version of the scale was developed in a sample of adults of both sexes, using exploratory factorial analysis. The univariate solution was formed by 10 of the 13 original items (items 1, 2, 3, 4, 5, 6, 7, 10, 11, and 13) [30]. The responses are scored on a 5-point Likert scale, ranging from Never = 1 to Always = 5. The sum of all items gives the final score. The higher the score, the greater the body appreciation.

*Common mental disorders–General Health Questionnaire*. The 12-item Brazilian version of the General Health Questionnaire, a screening tool for non-psychotic mental disorders, including anxiety, depression, and somatic symptoms, will be used to identify common mental disorders [31]. Each question has four response options, two considered negative (and scoring 0) and two considered positive (and scoring 1 each). For instance, the first item is: “*In the last two weeks*, *have you lost a lot of sleep through worry*?”. The response options are *Not at all*, *No more than usual*, *A little more than usual*, and *Much more than usual*. Participants will be classified as positive for common mental disorders if their overall score is 4 or more.

*Depression–patient health questionnaire*. This nine-item instrument examines for depressive episodes, using a four-point scale–from 0 (No day) to 3 (Almost every day)–to assess the frequency of signs and symptoms of depression in the prior two weeks. The sum of the item points gives the scores. For both males and females, values > 9 are considered to identify individuals at high risk of a major depressive episode [32].

#### Neighborhood satisfaction

Participants’ satisfaction with the neighborhood will be assessed through eight Yes/No questions developed by a research team of the Bambuí Cohort Study of a cohort of Brazilian older adults based on previous literature [33]: “*Do you feel comfortable in your neighborhood*, *that is*, *do you feel at home*?”; “*Are you satisfied with how your neighborhood is being taken care of*?”; “*Is your neighborhood a good place for you to live*? *Do you like your neighbors and your house*?”; “*Are you proud when you tell others where you live*?”; “*Would you like to move out of that neighborhood*?” (reverse-coded question to ensure data consistency); “*Are your neighbors willing to help each other*?”; “*Do children and young people in your neighborhood treat adults with respect*?”; “*Do you think your neighborhood is a good place for children to play; and a good place to raise teenagers*?”. A Cronbach’s alpha of 0.79 for psychometric indicators was found, indicating a high internal consistency. Each positive response adds one point to the final score.

#### Additional individual measurements

Sociodemographic information (age, sex, race, and marital status), self-reported physical morbidity, sedentary behavior, body weight, height, waist circumference, blood pressure, tobacco use, eating behavior, motivation for and tolerance of physical exercise, participation in physical education classes, and alcohol consumption will also be investigated. All anthropometric and blood pressure measurements will be taken following standard procedures [34, 35].

### Environmental measurements

#### Characterization and spatialization of the environment

**Fig 1:**
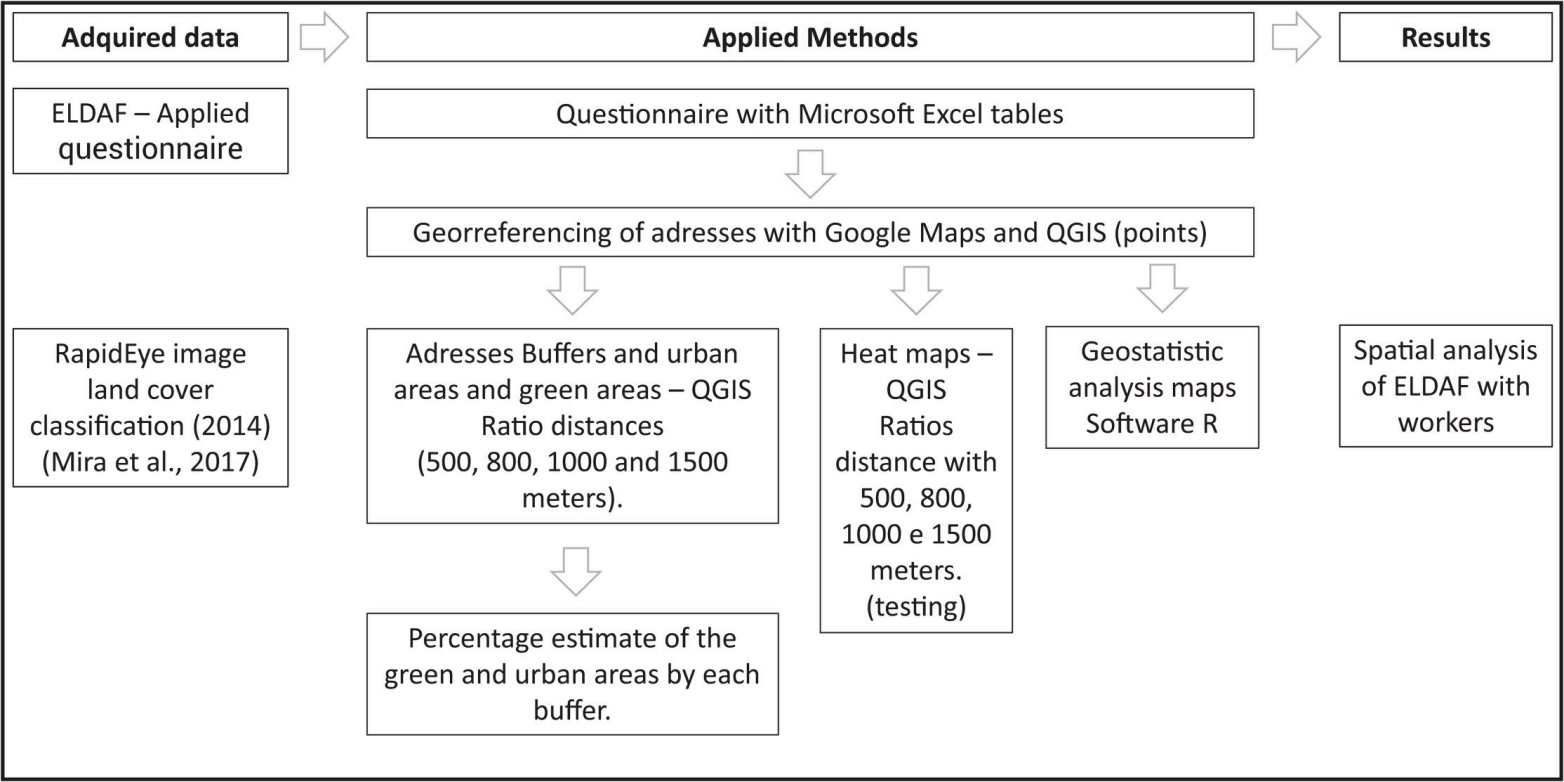
Methodological description of the georeferencing process.

Participants’ residences will be geocoded using residential postal codes. Geographical coordinates (latitude and longitude) will be obtained using participants’ complete addresses. Georeferenced information will be provided by the university and by participants in each phase. Neighborhoods were defined using road network buffers centered on participants’ geocoded residential addresses and calculated at four geographical scales commonly used in built environment and health research [4]: 500 m, 800 m, 1000 m, and 1500 m. The details of the entire process can be seen in Fig 1.

#### Public parks and squares

Georeferenced information of parks, public squares, and places for practicing physical activity, and population density will be used to create data about the built environment. Parks were defined as publicly owned, freely accessible, outdoor spaces intended for leisure or recreation activities. Accordingly, aquariums, golf courses, community centers, marinas, cemeteries, and similar places not located entirely within other public parks will be excluded. Data not stored in Geographic Information System (GIS) format will be digitized in QGIS 9.2 software. GIS park polygons were aggregated by unique park names for a final dataset of 1440 discrete parks. Additionally, the following question will be asked: “*Did you have any park or square close to your home*?”.

#### Green areas

Green areas are any areas around participants’ place of residence containing some vegetation cover, including trees on sidewalks, flowerbeds or squares, public or residential gardens, parks, or recreation facilities. Exposure will be estimated by the normalized difference vegetation index (NDVI). The NDVI, calculated using satellite remote sensing images, measures the vegetation density of a region. The index is determined by the difference between the two bands divided by the sum. Each pixel of the image receives an NDVI value ranging between 1 and -1 (in practice, values between -0.1 and 0.7). Negative values indicate water, ice, and clouds; values between -0.1 and 0.1 indicate uncovered surfaces, and around 0.6 show dense green vegetation [36].

### Quality assurance and control

Two pilot studies were performed to identify and correct potential problems in the data collection instruments and procedures. Applicators are trained and certified centrally following the study protocol. The ELDAF operation manuals specify standardized study procedures in detail. Periodic staff meetings will be held during data collection to discuss problems and check that standardized procedures are being correctly followed.

### Data analysis

Descriptive analysis will be performed to characterize the participants’ education level, morbidity, exposures, and outcomes. Although ELDAF focuses on the relationship between psychosocial/environmental factors and physical activity dimensions, the data analysis will depend on the theoretical model adopted for each approach.

### Ethical considerations and spread

Written informed consent will be obtained from all participants before the study begins. The *Universidade Salgado de Oliveira* research ethics committee has approved all procedures (approved by numbers 4.082.122 and CAAE: 56224716.2.0000.5289 in June 10, 2020). ELDAF also get a register in Open Science Framework (osf.io/nrsju). Data will be entered and stored in a secure database. Only a limited group of researchers will have access to the data. The study results will be published in peer-reviewed journals. In order to circulate the research findings to a broader audience, presentations will be made at international conferences and local workshops will be held at the university.

### Strengths and limitations of this study

Given that most scientific information regarding the middle-income countries is based on personal physical activity measurements, this study’s use of an accelerometer combined with a subjective validation scale will provide new findings in this field. Although ELDAF does include a broad range of workers, they are of higher socioeconomic status than their private market peers. On the other hand, it must be considered that socioeconomic levels in the region where participants live and work are low, which may attenuate the effects of socioeconomic differences common in Rio de Janeiro State. Another important aspect of the study approach is that it includes workers living in urban and rural areas, enhancing the possibility of findings without precedent in the literature, since evidence regarding the determinants of physical activity in rural areas is scarce. Finally, it is important to emphasis the longitudinal design aiming to follow the participants through several years. The possibility of observing life changes could provide relevant information that is currently lacking.
