# Correction: Psychosocial and environmental determinants of physical activity in a Brazilian public university employees—ELDAF: A prospective cohort study protocol

**DOI:** 10.1371/journal.pone.0271139

**Published:** 2022-07-05

**Authors:** Aldair J. Oliveira, Claudia S. Lopes, Geraldo de Albuquerque Maranhão Neto, Gustavo Mota de Sousa, Vitor Paravidino, Mikael Rostila, Mauro Felippe Felix Mediano, Rosane Harter Griep, Wesley Souza do Vale, Fabiane Frota da Rocha Morgado

The seventh author’s initials are indexed incorrectly in PubMed. The correct initials are: Mediano MFF.
